# Supplementary material for: Perspectives in the Development of Tools to Assess Vaccine Literacy
Source: Vaccines (Basel). 2024 Apr 16;12(4):422. doi: 10.3390/vaccines12040422 (PMC11054371; doi:10.3390/vaccines12040422)
Supplement: Supplementary file 1 [file vaccines-12-00422-s001.zip › vaccines-2885261-supplementary.pdf]

Development of Tools to Assess Vaccine Literacy: a Scoping Review and Future Perspectives
Supplementary material

Variables' psychological glossary considered for building the framework

Term	APA definition (https://www.apa.org/)	Common definitions reported in literature
**Ability**	existing competence or skill to perform a specific physical or mental act. Although ability may be either innate or developed through experience, it is distinct from capacity to acquire competence	innate potential to perform mental and physical actions or tasks
**Attitude**	relatively enduring and general evaluation of an object, person, group, issue, or concept on a dimension ranging from negative to positive. Attitudes provide summary evaluations of target objects and are often assumed to be derived from specific beliefs, emotions, and past behaviors	internal construct within an individual's mind which reflects thoughts, beliefs, emotions and evaluation
**Behavior**	any action or function that can be objectively observed in response to controlled stimuli.	external manifestation that can be observed by others and reflects an individual's actions in the external world
**Belief**	acceptance of the truth, reality, or validity of something (e.g., a person's veracity), particularly in the absence of substantiation.	descriptive thought that persons hold about something
**Capacity**	maximum ability of an individual to receive or retain information and knowledge or to function in mental or physical tasks.	an individual's mental or physical ability
**Competencies**	-	knowledge, skills, abilities, and behaviors that contribute to individual and organizational performance
**Emotion**	complex reaction pattern, involving experiential, behavioral, and physiological elements, by which an individual attempts to deal with a personally significant matter or event. Emotion typically involves feeling but differs from feeling in having an overt or implicit engagement with the world	strong feeling deriving from one's circumstances, mood, or relationships with others
**Feeling**	self-contained phenomenal experience, subjective, evaluative, and independent of the sensations, thoughts, or images evoking it Feelings differ from emotions in being purely mental, whereas emotions are designed to engage with the world	subjective, evaluative, and independent of the sensations, thought
**Knowledge**	the state of being familiar with something or aware of its existence, usually resulting from experience or study	information learned through experience, study or investigation
**Motivation**	a person's willingness to exert physical or mental effort in pursuit of a goal or outcome	internal and external: process that initiates, guides, and maintains goal-oriented behaviors
**Perception**	the process or result of becoming aware of objects, relationships, and events by means of the senses, which including such activities as recognizing, observing, and discriminating	
**Self-efficacy**	an individual's subjective perception of their capability to perform in a given setting or to attain desired results, proposed by Albert Bandura as a primary determinant of emotional and motivational states and behavioral change.	individual's belief in their own ability to successfully complete tasks, achieve goals, and handle challenges
**Skill**	an ability or proficiency acquired through training and practice.	the result of repeatedly applying knowledge or ability

GLM MEDIATION MODEL applied on the data set of the 2020 survey (N=885);
Gallucci, M. (2020). jAMM: jamovi Advanced Mediation Models.- https://jamovi-amm.github

LEGENDA OF ITEMS INTRODUCED IN THE MEDIATION MODEL AND PCA OF 2020 SURVEY DATA (N=885)[17]

**FUVL ITEMS - When reading or listening to information about future Covid-19 vaccines or current vaccines:**
ITEM1= Did you find words you didn't know?
ITEM2= Did you find that the texts were difficult to understand?
ITEM3= Did you need much time to understand them?
ITEM4= Did you or would you need someone to help you understand them?
FUVLNOM= Functional VL nominal scale
**ICVL ITEMS - When looking for information about future Covid-19 vaccines or current vaccines:**
ITEM5= Have you consulted more than one source of information?
ITEM 6= Did you find the information you were looking for?
ITEM 7= Have you had the opportunity to use the information?
ITEM 8= Did you discuss what you understood about vaccinations with your doctor or other people?
ITEM 9= Did you consider whether the information collected was about your condition?
ITEM 10= Have you considered the credibility of the sources?
ITEM 11= Did you check whether the information was correct?
ITEM 12= Did you find any useful information to make a decision on whether or not to get vaccinated?
ICVLNOM= Interactive-critical VL nominal scale
**BELIEFS ABOUT VACCINATION – about general vaccination**
1) BEL1EF1 / **CONF IDENCE** - 'I am not favorable to vaccines because they are unsafe'
2) BELIEF2 / **COMP LACENCY** - 'There is no need to vaccinate because natural immunity exists'
**Attitudes (QUEstions) – ABOUT Covid-19 vaccines**
1) QUE1 Will be possible to produce safe and efficacious vaccines?
2) QUE2 / **INT ENTION** - Will you get vaccinated, if possible?
3) QUE3 Will Health Authorities succeed in vaccinating the entire population?
4) QUE4 / **CONV ENIENCE** - Would you pay a fee to be vaccinated?
5) QUE5CHI -Should children be vaccinated too?
**Demographics and outcome**
AGE CLASS = Four classes: 1=18-30 - 2=31-50 - 3=51-65 - 4=>65 years of age
EDUCATION = Four level:: 1= primary – 2= secondary – 3= tertiary – 4= master
FLULAST = last seasonal flu vaccine received (uptake) yes / no

VL mediating effects between antecedents and '3Cs' - Indirect and Total Effects **C.I. 95%** *computed with method: Standard (Delta method)*

Type	DETERMINANT > VL > CONFIDENCE	Estimate	SE	Lower	Upper	β	z	p	%
Indirect	EDUCATION ⇒ FUVL ⇒ CONFIDENCE	0,00589	0,00275	4,97E-04	0,01128	0,01083	2.141	0,032	**0,11**
	EDUCATION ⇒ ICVL ⇒ CONFIDENCE	0,01744	0,00474	0,00815	0,02674	0,03207	3.678	<,001	**0,32**
	AGECLASS ⇒ FUVL ⇒ CONFIDENCE	-7,27e−4	0,00199	-0,00463	0,00318	-0,00121	-0,365	0,715	
	AGECLASS ⇒ ICVL ⇒ CONFIDENCE	0,00747	0,00401	-3,95e−4	0,01533	0,01241	1.861	0,063	
	HCWORKER ⇒ FUVL ⇒ CONFIDENCE	0,02122	0,009	0,00359	0,03886	0,01372	2.358	0,018	
	HCWORKER ⇒ ICVL ⇒ CONFIDENCE	0,02738	0,01116	0,0055	0,04927	0,0177	2.453	0,014	
Component	EDUCATION ⇒ FUVL	0,07644	0,0241	0,0292	0,12368	0,11032	3.171	0,002	
	FUVL ⇒ CONFIDENCE	0,07706	0,02656	0,02501	0,12912	0,09818	2.902	0,004	
	EDUCATION ⇒ ICVL	0,08932	0,01817	0,05371	0,12492	0,16982	4.917	<,001	
	ICVL ⇒ CONFIDENCE	0,1953	0,03524	0,12623	0,26437	0,18887	5.542	<,001	
	AGECLASS ⇒ FUVL	-0,00944	0,02566	-0,05974	0,04086	-0,01231	-0,368	0,713	
	AGECLASS ⇒ ICVL	0,03823	0,01934	3,13E-04	0,07614	0,0657	1.976	0,048	
	HCWORKER ⇒ FUVL	0,27539	0,06802	0,14208	0,4087	0,13971	4.049	<,001	
	HCWORKER ⇒ ICVL	0,14021	0,05127	0,03973	0,24068	0,0937	2.735	0,006	
Direct	EDUCATION ⇒ CONFIDENCE	0,03095	0,01897	-0,00623	0,06813	0,05692	1.632	0,103	
	AGECLASS ⇒ CONFIDENCE	-0,04656	0,01991	-0,08558	-0,00754	-0,0774	-2.339	0,019	
	HCWORKER ⇒ CONFIDENCE	0,03275	0,05326	-0,07164	0,13714	0,02117	0,615	0,539	
Total	EDUCATION ⇒ CONFIDENCE	0,05429	0,01914	0,01677	0,09181	0,09982	2.836	0,005	
	AGECLASS ⇒ CONFIDENCE	-0,03982	0,02038	-0,07977	1,26E-04	-0,0662	-1.954	0,051	
	HCWORKER ⇒ CONFIDENCE	0,08135	0,05402	-0,02452	0,18723	0,05258	1.506	0,132	
	**DETERMINANT > VL > COMPLACENCY**								
Indirect	EDUCATION ⇒ FUVL ⇒ COMPLACENCY	0,00195	0,00211	-0,0022	0,00609	0,00366	0,921	0,357	
	EDUCATION ⇒ ICVL ⇒ COMPLACENCY	0,01208	0,00398	0,00427	0,01989	0,02272	3.033	0,002	**0,25**
	AGECLASS ⇒ FUVL ⇒ COMPLACENCY	-2,40e−4	6,99E-04	-0,00161	0,00113	-4,08e−4	-0,343	0,731	
	AGECLASS ⇒ ICVL ⇒ COMPLACENCY	0,00517	0,00294	-5,93e−4	0,01094	0,00879	1.758	0,079	
	HCWORKER ⇒ FUVL ⇒ COMPLACENCY	0,00701	0,00749	-0,00767	0,02169	0,00463	0,936	0,349	
	HCWORKER ⇒ ICVL ⇒ COMPLACENCY	0,01897	0,0085	0,0023	0,03564	0,01254	2.230	0,026	
Component	EDUCATION ⇒ FUVL	0,07644	0,0241	0,0292	0,12368	0,11032	3.171	0,002	
	FUVL ⇒ COMPLACENCY	0,02546	0,02646	-0,02641	0,07732	0,03317	0,962	0,336	
	EDUCATION ⇒ ICVL	0,08932	0,01817	0,05371	0,12492	0,16982	4.917	<,001	
	ICVL ⇒ COMPLACENCY	0,13528	0,03511	0,06647	0,20409	0,13378	3.853	<,001	
	AGECLASS ⇒ FUVL	-0,00944	0,02566	-0,05974	0,04086	-0,01231	-0,368	0,713	
	AGECLASS ⇒ ICVL	0,03823	0,01934	3,13E-04	0,07614	0,0657	1.976	0,048	
	HCWORKER ⇒ FUVL	0,27539	0,06802	0,14208	0,4087	0,13971	4.049	<,001	
	HCWORKER ⇒ ICVL	0,14021	0,05127	0,03973	0,24068	0,0937	2.735	0,006	
Direct	EDUCATION ⇒ COMPLACENCY	0,03454	0,0189	-0,0025	0,07159	0,06495	1.828	0,068	
	AGECLASS ⇒ COMPLACENCY	0,00567	0,01984	-0,03321	0,04455	0,00964	0,286	0,775	
	HCWORKER ⇒ COMPLACENCY	0,02687	0,05306	-0,07713	0,13088	0,01776	0,506	0,613	
Total	EDUCATION ⇒ COMPLACENCY	0,04857	0,01878	0,01177	0,08538	0,09133	2.587	0,01	
	AGECLASS ⇒ COMPLACENCY	0,0106	0,02	-0,02859	0,04979	0,01802	0,53	0,596	
	HCWORKER ⇒ COMPLACENCY	0,05285	0,05299	-0,05101	0,15671	0,03493	0,997	0,319	
	**DETERMINANT > VL > CONVENIENCE**								
Indirect	EDUCATION ⇒ FUVL ⇒ CONVENIENCE	-6,82e−4	0,0014	-0,00342	0,00206	-0,00188	-0,488	0,626	
	EDUCATION ⇒ ICVL ⇒ CONVENIENCE	0,01103	0,0031	0,00495	0,01711	0,03037	3.557	<,001	**0,57**
	AGECLASS ⇒ FUVL ⇒ CONVENIENCE	8,42E-05	2,85E-04	-4,75e−4	6,44E-04	2,09E-04	0,295	0,768	
	AGECLASS ⇒ ICVL ⇒ CONVENIENCE	0,00472	0,00256	-2,94e−4	0,00974	0,01175	1.845	0,065	
	HCWORKER ⇒ FUVL ⇒ CONVENIENCE	-0,00246	0,00501	-0,01228	0,00737	-0,00238	-0,49	0,624	
	HCWORKER ⇒ ICVL ⇒ CONVENIENCE	0,01732	0,00717	0,00327	0,03137	0,01676	2.416	0,016	
Component	EDUCATION ⇒ FUVL	0,07644	0,0241	0,0292	0,12368	0,11032	3.171	0,002	
	FUVL ⇒ CONVENIENCE	-0,00892	0,01806	-0,04432	0,02649	-0,01701	-0,494	0,621	
	EDUCATION ⇒ ICVL	0,08932	0,01817	0,05371	0,12492	0,16982	4.917	<,001	
	ICVL ⇒ CONVENIENCE	0,12351	0,02397	0,07654	0,17049	0,17885	5.154	<,001	
	AGECLASS ⇒ FUVL	-0,00944	0,02566	-0,05974	0,04086	-0,01231	-0,368	0,713	
	AGECLASS ⇒ ICVL	0,03823	0,01934	3,13E-04	0,07614	0,0657	1.976	0,048	
	HCWORKER ⇒ FUVL	0,27539	0,06802	0,14208	0,4087	0,13971	4.049	<,001	
	HCWORKER ⇒ ICVL	0,14021	0,05127	0,03973	0,24068	0,0937	2.735	0,006	
Direct	EDUCATION ⇒ CONVENIENCE	0,00884	0,0129	-0,01645	0,03413	0,02434	0,685	0,493	
	AGECLASS ⇒ CONVENIENCE	-0,00225	0,01354	-0,02879	0,02429	-0,00561	-0,166	0,868	
	HCWORKER ⇒ CONVENIENCE	-0,03548	0,03622	-0,10648	0,03551	-0,03434	-0,98	0,327	
Total	EDUCATION ⇒ CONVENIENCE	0,01919	0,01289	-0,00606	0,04445	0,05284	1.489	0,136	
	AGECLASS ⇒ CONVENIENCE	0,00255	0,01372	-0,02434	0,02944	0,00636	0,186	0,852	
	HCWORKER ⇒ CONVENIENCE	-0,02062	0,03636	-0,09189	0,05064	-0,01996	-0,567	0,571	

**'3Cs' mediating effects between VL and outcome - Indirect and Total Effects -**		**C.I. 95%** ***computed with method: Standard (Delta method)***							
Type	VL > CONFIDENCE > FLU VACCINE UPTAKE	**Estimate**	**SE**	**Lower**	**Upper**	**β**	**z**	**p**	**%**
Indirect	FUVL ⇒ CONFIDENCE ⇒ FLULAST	0,0114	0,00448	0,00262	0,02018	0,0163	2.545	0,011	**0,14**
	ICVL ⇒ CONFIDENCE ⇒ FLULAST	0,02889	0,00754	0,01411	0,04367	0,03137	3.832	<,001	**0,37**
	EDUCATION ⇒ CONFIDENCE ⇒ FLULAST	0,00458	0,00294	-0,00118	0,01033	0,00945	1.560	0,119	
	AGECLASS ⇒ CONFIDENCE ⇒ FLULAST	-0,00689	0,00322	-0,0132	-5,79e−4	-0,01285	-2.140	0,032	
	HCWORKER ⇒ CONFIDENCE ⇒ FLULAST	0,00484	0,00793	-0,0107	0,02039	0,00351	0,611	0,541	
Component	FUVL ⇒ CONFIDENCE	0,07706	0,02656	0,02501	0,12912	0,09818	2.902	0,004	
	CONFIDENCE ⇒ FLULAST	0,14792	0,02789	0,09325	0,20259	0,16607	5.303	<,001	
	ICVL ⇒ CONFIDENCE	0,1953	0,03524	0,12623	0,26437	0,18887	5.542	<,001	
	EDUCATION ⇒ CONFIDENCE	0,03095	0,01897	-0,00623	0,06813	0,05692	1.632	0,103	
	AGECLASS ⇒ CONFIDENCE	-0,04656	0,01991	-0,08558	-0,00754	-0,0774	-2.339	0,019	
	HCWORKER ⇒ CONFIDENCE	0,03275	0,05326	-0,07164	0,13714	0,02117	0,615	0,539	
Direct	FUVL ⇒ FLULAST	0,06729	0,02197	0,02423	0,11035	0,09624	3.063	0,002	
	ICVL ⇒ FLULAST	0,04827	0,02952	-0,00958	0,10612	0,05241	1.635	0,102	
	EDUCATION ⇒ FLULAST	0,01409	0,01564	-0,01656	0,04475	0,02909	0,901	0,368	
	AGECLASS ⇒ FLULAST	0,18136	0,01644	0,14913	0,21358	0,33846	11.031	<,001	
	HCWORKER ⇒ FLULAST	0,20187	0,04385	0,11592	0,28782	0,14648	4.603	<,001	
Total	FUVL ⇒ FLULAST	0,07869	0,02223	0,03512	0,12225	0,11254	3.540	<,001	
	ICVL ⇒ FLULAST	0,07716	0,02949	0,01936	0,13496	0,08378	2.616	0,009	
	EDUCATION ⇒ FLULAST	0,01867	0,01588	-0,01244	0,04979	0,03854	1.176	0,24	
	AGECLASS ⇒ FLULAST	0,17447	0,01666	0,14181	0,20713	0,32561	10.471	<,001	
	HCWORKER ⇒ FLULAST	0,20672	0,04457	0,11936	0,29408	0,15	4.638	<,001	
**VL > COMPLACENCY > FLU VACCINE UPTAKE**									
Indirect	FUVL ⇒ COMPLACENCY ⇒ FLULAST	0,00376	0,00397	-0,00402	0,01154	0,00537	0,946	0,344	
	ICVL ⇒ COMPLACENCY ⇒ FLULAST	0,01997	0,00642	0,00739	0,03255	0,02168	3.111	0,002	**0,26**
	EDUCATION ⇒ COMPLACENCY ⇒ FLULAST	0,0051	0,00295	-6,89e−4	0,01088	0,01052	1.727	0,084	
	AGECLASS ⇒ COMPLACENCY ⇒ FLULAST	8,37E-04	0,00293	-0,00491	0,00658	0,00156	0,285	0,775	
	HCWORKER ⇒ COMPLACENCY ⇒ FLULAST	0,00397	0,00787	-0,01145	0,01939	0,00288	0,504	0,614	
Component	FUVL ⇒ COMPLACENCY	0,02546	0,02646	-0,02641	0,07732	0,03317	0,962	0,336	
	COMPLACENCY ⇒ FLULAST	0,14759	0,028	0,09271	0,20247	0,16204	5.271	<,001	
	ICVL ⇒ COMPLACENCY	0,13528	0,03511	0,06647	0,20409	0,13378	3.853	<,001	
	EDUCATION ⇒ COMPLACENCY	0,03454	0,0189	-0,0025	0,07159	0,06495	1.828	0,068	
	AGECLASS ⇒ COMPLACENCY	0,00567	0,01984	-0,03321	0,04455	0,00964	0,286	0,775	
	HCWORKER ⇒ COMPLACENCY	0,02687	0,05306	-0,07713	0,13088	0,01776	0,506	0,613	
Direct	FUVL ⇒ FLULAST	0,07493	0,02188	0,03204	0,11781	0,10717	3.425	<,001	
	ICVL ⇒ FLULAST	0,05719	0,02926	-1,55e−4	0,11454	0,0621	1.955	0,051	
	EDUCATION ⇒ FLULAST	0,01357	0,01565	-0,0171	0,04424	0,02802	0,867	0,386	
	AGECLASS ⇒ FLULAST	0,17363	0,01639	0,1415	0,20576	0,32404	10.592	<,001	
	HCWORKER ⇒ FLULAST	0,20275	0,04386	0,11679	0,28871	0,14712	4.623	<,001	
Total	FUVL ⇒ FLULAST	0,07869	0,02223	0,03512	0,12225	0,11254	3.540	<,001	
	ICVL ⇒ FLULAST	0,07716	0,02949	0,01936	0,13496	0,08378	2.616	0,009	
	EDUCATION ⇒ FLULAST	0,01867	0,01588	-0,01244	0,04979	0,03854	1.176	0,24	
	AGECLASS ⇒ FLULAST	0,17447	0,01666	0,14181	0,20713	0,32561	10.471	<,001	
	HCWORKER ⇒ FLULAST	0,20672	0,04457	0,11936	0,29408	0,15	4.638	<,001	
**VL > CONVENIENCE > FLU VACCINE UPTAKE**									
Indirect	FUVL ⇒ CONVENIENCE ⇒ FLULAST	-0,00182	0,0037	-0,00906	0,00543	-0,0026	-0,491	0,623	
	ICVL ⇒ CONVENIENCE ⇒ FLULAST	0,02516	0,00704	0,01136	0,03896	0,02732	3.573	<,001	**0,33**
	EDUCATION ⇒ CONVENIENCE ⇒ FLULAST	0,0018	0,00265	-0,0034	0,007	0,00372	0,679	0,497	
	AGECLASS ⇒ CONVENIENCE ⇒ FLULAST	-4,59e−4	0,00276	-0,00587	0,00495	-8,56e−4	-0,166	0,868	
	HCWORKER ⇒ CONVENIENCE ⇒ FLULAST	-0,00723	0,00752	-0,02197	0,00751	-0,00525	-0,961	0,337	
Component	FUVL ⇒ CONVENIENCE	-0,00892	0,01806	-0,04432	0,02649	-0,01701	-0,494	0,621	
	CONVENIENCE ⇒ FLULAST	0,20371	0,04109	0,12317	0,28425	0,15275	4.957	<,001	
	ICVL ⇒ CONVENIENCE	0,12351	0,02397	0,07654	0,17049	0,17885	5.154	<,001	
	EDUCATION ⇒ CONVENIENCE	0,00884	0,0129	-0,01645	0,03413	0,02434	0,685	0,493	
	AGECLASS ⇒ CONVENIENCE	-0,00225	0,01354	-0,02879	0,02429	-0,00561	-0,166	0,868	
	HCWORKER ⇒ CONVENIENCE	-0,03548	0,03622	-0,10648	0,03551	-0,03434	-0,98	0,327	
Direct	FUVL ⇒ FLULAST	0,0805	0,02191	0,03756	0,12345	0,11514	3.674	<,001	
	ICVL ⇒ FLULAST	0,052	0,02951	-0,00583	0,10983	0,05646	1.762	0,078	
	EDUCATION ⇒ FLULAST	0,01687	0,01565	-0,01381	0,04755	0,03483	1.078	0,281	
	AGECLASS ⇒ FLULAST	0,17493	0,01642	0,14274	0,20712	0,32646	10.652	<,001	
	HCWORKER ⇒ FLULAST	0,21395	0,04395	0,1278	0,3001	0,15524	4.867	<,001	
Total	FUVL ⇒ FLULAST	0,07869	0,02223	0,03512	0,12225	0,11254	3.540	<,001	
	ICVL ⇒ FLULAST	0,07716	0,02949	0,01936	0,13496	0,08378	2.616	0,009	
	EDUCATION ⇒ FLULAST	0,01867	0,01588	-0,01244	0,04979	0,03854	1.176	0,24	
	AGECLASS ⇒ FLULAST	0,17447	0,01666	0,14181	0,20713	0,32561	10.471	<,001	
	HCWORKER ⇒ FLULAST	0,20672	0,04457	0,11936	0,29408	0,15	4.638	<,001	

**Single VL ITEMS mediating effects between 'education' and '3Cs'**		**Indirect and Total Effects**							
Confidence intervals computed with method: Standard (Delta method)									
				**95% C.I.**					
**Type**	**Effect**	**Estimate**	**SE**	**Lower**	**Upper**	**β**	**z**	**P**	**%**
Indirect	EDUCAT ⇒ ITEM1 ⇒ CONFIDENCE	0,00591	0,00441	-0,0027	0,01456	0,01088	1.339	0,181	**0,10**
	EDUCAT ⇒ ITEM2 ⇒ CONFIDENCE	0,00542	0,0039	-0,0022	0,01306	0,00998	1.391	0,164	**0,09**
	EDUCAT ⇒ ITEM3 ⇒ CONFIDENCE	0,00319	0,00287	-0,0024	0,00882	0,00587	1.110	0,267	**0,06**
	EDUCAT ⇒ ITEM4 ⇒ CONFIDENCE	-0,0011	0,00206	-0,0052	0,00291	-0,0021	-0,546	0,585	**-0,02**
Component	EDUCAT ⇒ ITEM1	0,15063	0,02692	0,09786	0,2034	0,18483	5.595	< ,001	
	ITEM1 ⇒ CONFIDENCE	0,03922	0,02844	-0,0165	0,09496	0,05886	1.379	0,168	
	EDUCAT ⇒ ITEM2	0,10542	0,02577	0,0549	0,15593	0,1362	4.090	< ,001	
	ITEM2 ⇒ CONFIDENCE	0,05142	0,03477	-0,0167	0,11956	0,07329	1.479	0,139	
	EDUCAT ⇒ ITEM3	0,08004	0,02788	0,02539	0,1347	0,09605	2.871	0,004	
	ITEM3 ⇒ CONFIDENCE	0,03983	0,03308	-0,025	0,10467	0,06114	1.204	0,229	
	EDUCAT ⇒ ITEM4	0,0725	0,0303	0,01312	0,13188	0,08018	2.393	0,017	
	ITEM4 ⇒ CONFIDENCE	-0,0155	0,02768	-0,0698	0,03873	-0,0259	-0,561	0,575	
Direct	EDUCAT ⇒ CONFIDENCE	0,04413	0,01828	0,0083	0,07996	0,08128	2.414	0,016	
Total	EDUCAT ⇒ CONFIDENCE	0,05752	0,01816	0,02193	0,09311	0,10594	3.168	0,002	
**Type**	**Effect**	**Estimate**	**SE**	**Lower**	**Upper**	**β**	**z**	**P**	**%**
Indirect	EDUCAT ⇒ ITEM5 ⇒ CONFIDENCE	1,38E-04	0,00129	-0,0024	0,00266	2,54E-04	0,107	0,914	**0,00**
	EDUCAT ⇒ ITEM6 ⇒ CONFIDENCE	0,00248	0,002	-0,0014	0,0064	0,00457	1.239	0,215	**0,04**
	EDUCAT ⇒ ITEM7 ⇒ CONFIDENCE	0,00396	0,00293	-0,0018	0,0097	0,0073	1.354	0,176	**0,07**
	EDUCAT ⇒ ITEM8 ⇒ CONFIDENCE	-4,76e−4	0,00351	-0,0074	0,00641	-8,77e−4	-0,136	0,892	**na**
	EDUCAT ⇒ ITEM9 ⇒ CONFIDENCE	-0,0073	0,00313	-0,0134	-0,0011	-0,0134	-2.323	0,02	**-0,13**
	EDUCAT ⇒ ITEM10 ⇒ CONFIDENCE	0,01163	0,00404	0,00372	0,01955	0,02143	2.882	0,004	**0,20**
	EDUCAT ⇒ ITEM11 ⇒ CONFIDENCE	0,00262	0,0032	-0,0036	0,00889	0,00483	0,821	0,412	**0,05**
	EDUCAT ⇒ ITEM12 ⇒ CONFIDENCE	0,01136	0,00405	0,00342	0,01929	0,02091	2.805	0,005	**0,20**
Component	EDUCAT ⇒ ITEM5	0,0531	0,02822	-0,0022	0,10842	0,06312	1.882	0,06	
	ITEM5 ⇒ CONFIDENCE	0,0026	0,02417	-0,0448	0,04996	0,00403	0,108	0,914	
	EDUCAT ⇒ ITEM6	0,05943	0,02473	0,01096	0,10789	0,08051	2.403	0,016	
	ITEM6 ⇒ CONFIDENCE	0,04175	0,02886	-0,0148	0,09832	0,05675	1.447	0,148	
	EDUCAT ⇒ ITEM7	0,08824	0,02462	0,03999	0,1365	0,11963	3.584	< ,001	
	ITEM7 ⇒ CONFIDENCE	0,0449	0,0307	-0,0153	0,10507	0,061	1.463	0,144	
	EDUCAT ⇒ ITEM8	0,18483	0,03462	0,11698	0,25269	0,17665	5.339	< ,001	
	ITEM8 ⇒ CONFIDENCE	-0,0026	0,01899	-0,0398	0,03465	-0,005	-0,136	0,892	
	EDUCAT ⇒ ITEM9	0,13794	0,03494	0,06946	0,20641	0,13156	3.948	< ,001	
	ITEM9 ⇒ CONFIDENCE	-0,0528	0,01836	-0,0888	-0,0168	-0,1019	-2.873	0,004	
	EDUCAT ⇒ ITEM10	0,09574	0,0191	0,05831	0,13318	0,16615	5.013	< ,001	
	ITEM10 ⇒ CONFIDENCE	0,12151	0,0345	0,0539	0,18912	0,12896	3.522	< ,001	
	EDUCAT ⇒ ITEM11	0,11658	0,02717	0,06333	0,16982	0,14277	4.291	< ,001	
	ITEM11 ⇒ CONFIDENCE	0,02251	0,02693	-0,0303	0,07529	0,03386	0,836	0,403	
	EDUCAT ⇒ ITEM12	0,13187	0,03124	0,07064	0,19309	0,14049	4.221	< ,001	
	ITEM12 ⇒ CONFIDENCE	0,08611	0,02294	0,04115	0,13107	0,14886	3.754	< ,001	
Direct	EDUCAT ⇒ CONFIDENCE	0,03308	0,01793	-0,0021	0,06822	0,06092	1.845	0,065	
Total	EDUCAT ⇒ CONFIDENCE	0,05752	0,01816	0,02193	0,09311	0,10594	3.168	0,002	
**Type**	**Effect**	**Estimate**	**SE**	**Lower**	**Upper**	**β**	**z**	**P**	**%**
Indirect	EDUCAT ⇒ ITEM1 ⇒ COMPLACENCY	0,00232	0,00423	-0,006	0,01061	0,00438	0,549	0,583	**0,04**
	EDUCAT ⇒ ITEM2 ⇒ COMPLACENCY	0,00836	0,00414	2,43E-04	0,01647	0,01577	2.019	0,044	**0,16**
	EDUCAT ⇒ ITEM3 ⇒ COMPLACENCY	-4,48e−4	0,00261	-0,0056	0,00466	-8,46e−4	-0,172	0,863	**na**
	EDUCAT ⇒ ITEM4 ⇒ COMPLACENCY	-0,0024	0,00221	-0,0067	0,00195	-0,0045	-1.077	0,281	**-0,04**
Component	EDUCAT ⇒ ITEM1	0,15063	0,02692	0,09786	0,2034	0,18483	5.595	< ,001	
	ITEM1 ⇒ COMPLACENCY	0,01541	0,02794	-0,0394	0,07018	0,0237	0,552	0,581	
	EDUCAT ⇒ ITEM2	0,10542	0,02577	0,0549	0,15593	0,1362	4.090	< ,001	
	ITEM2 ⇒ COMPLACENCY	0,07928	0,03416	0,01234	0,14622	0,11579	2.321	0,02	
	EDUCAT ⇒ ITEM3	0,08004	0,02788	0,02539	0,1347	0,09605	2.871	0,004	
	ITEM3 ⇒ COMPLACENCY	-0,0056	0,0325	-0,0693	0,0581	-0,0088	-0,172	0,863	
	EDUCAT ⇒ ITEM4	0,0725	0,0303	0,01312	0,13188	0,08018	2.393	0,017	
	ITEM4 ⇒ COMPLACENCY	-0,0328	0,02719	-0,0861	0,02048	-0,056	-1.207	0,228	
Direct	EDUCAT ⇒ COMPLACENCY	0,04528	0,01796	0,01008	0,08048	0,08544	2.521	0,012	
Total	EDUCAT ⇒ COMPLACENCY	0,05313	0,01773	0,01837	0,08789	0,10026	2.996	0,003	
**Type**	**Effect**	**Estimate**	**SE**	**Lower**	**Upper**	**β**	**z**	**P**	**%**
Indirect	EDUCAT ⇒ ITEM5 ⇒ COMPLACENCY	6,52E-04	0,00132	-0,0019	0,00324	0,00123	0,493	0,622	**0,01**
	EDUCAT ⇒ ITEM6 ⇒ COMPLACENCY	0,00137	0,0018	-0,0022	0,0049	0,00259	0,764	0,445	**0,03**
	EDUCAT ⇒ ITEM7 ⇒ COMPLACENCY	0,00404	0,00292	-0,0017	0,00976	0,00762	1.384	0,166	**0,08**
	EDUCAT ⇒ ITEM8 ⇒ COMPLACENCY	-0,0016	0,0035	-0,0084	0,00529	-0,003	-0,45	0,652	**-0,03**
	EDUCAT ⇒ ITEM9 ⇒ COMPLACENCY	-0,0044	0,00276	-0,0098	9,87E-04	-0,0083	-1.602	0,109	**-0,08**
	EDUCAT ⇒ ITEM10 ⇒ COMPLACENCY	0,01258	0,00413	0,00448	0,02068	0,02374	3.044	0,002	**0,24**
	EDUCAT ⇒ ITEM11 ⇒ COMPLACENCY	-0,0043	0,00328	-0,0108	0,0021	-0,0082	-1.319	0,187	**-0,08**
	EDUCAT ⇒ ITEM12 ⇒ COMPLACENCY	0,00872	0,00365	0,00157	0,01587	0,01646	2.390	0,017	**0,16**
Component	EDUCAT ⇒ ITEM5	0,0531	0,02822	-0,0022	0,10842	0,06312	1.881	0,06	
	ITEM5 ⇒ COMPLACENCY	0,01228	0,02402	-0,0348	0,05937	0,0195	0,511	0,609	

	EDUCAT ⇒ ITEM6	0,05943	0,02473	0,01096	0,10789	0,08051	2.403	0,016	
	ITEM6 ⇒ COMPLACENCY	0,02313	0,02869	-0,0331	0,07937	0,03222	0,806	0,42	
	EDUCAT ⇒ ITEM7	0,08824	0,02462	0,03999	0,1365	0,11963	3.584	<,001	
	ITEM7 ⇒ COMPLACENCY	0,04577	0,03052	-0,014	0,10559	0,06371	1.500	0,134	
	EDUCAT ⇒ ITEM8	0,18483	0,03462	0,11698	0,25269	0,17665	5.339	<,001	
	ITEM8 ⇒ COMPLACENCY	-0,0085	0,01888	-0,0455	0,02847	-0,0169	-0,452	0,651	
	EDUCAT ⇒ ITEM9	0,13794	0,03494	0,06946	0,20641	0,13156	3.948	<,001	
	ITEM9 ⇒ COMPLACENCY	-0,032	0,01826	-0,0678	0,00379	-0,0633	-1.752	0,08	
	EDUCAT ⇒ ITEM10	0,09574	0,0191	0,05831	0,13318	0,16615	5.013	<,001	
	ITEM10 ⇒ COMPLACENCY	0,13142	0,03429	0,0642	0,19864	0,1429	3.832	<,001	
	EDUCAT ⇒ ITEM11	0,11658	0,02717	0,06333	0,16982	0,14277	4.291	<,001	
	ITEM11 ⇒ COMPLACENCY	-0,0371	0,02677	-0,0896	0,01536	-0,0572	-1.386	0,166	
	EDUCAT ⇒ ITEM12	0,13187	0,03124	0,07064	0,19309	0,14049	4.221	<,001	
	ITEM12 ⇒ COMPLACENCY	0,06613	0,0228	0,02144	0,11083	0,11714	2.900	0,004	
Direct	EDUCAT ⇒ COMPLACENCY	0,03608	0,01783	0,00114	0,07101	0,06808	2.024	0,043	
Total	EDUCAT ⇒ COMPLACENCY	0,05313	0,01773	0,01837	0,08789	0,10026	2.996	0,003	
**Type**	**Effect**	**Estimate**	**SE**	**Lower**	**Upper**	**β**	**z**	**P**	**%**
Indirect	EDUCAT ⇒ ITEM1 ⇒ CONVENIENCE	0,00355	0,00299	-0,0023	0,0094	0,00973	1.190	0,234	**0,19**
	EDUCAT ⇒ ITEM2 ⇒ CONVENIENCE	0,00102	0,00251	-0,0039	0,00594	0,00281	0,409	0,683	**0,05**
	EDUCAT ⇒ ITEM3 ⇒ CONVENIENCE	0,00149	0,00188	-0,0022	0,00516	0,00407	0,792	0,428	**0,08**
	EDUCAT ⇒ ITEM4 ⇒ CONVENIENCE	-0,0031	0,00188	-0,0068	5,93E-04	-0,0085	-1.644	0,1	**-0,16**
Component	EDUCAT ⇒ ITEM1	0,15063	0,02692	0,09786	0,2034	0,18483	5.595	<,001	
	ITEM1 ⇒ CONVENIENCE	0,02359	0,01937	-0,0144	0,06155	0,05263	1.218	0,223	
	EDUCAT ⇒ ITEM2	0,10542	0,02577	0,0549	0,15593	0,1362	4.090	<,001	
	ITEM2 ⇒ CONVENIENCE	0,00972	0,02367	-0,0367	0,05612	0,0206	0,411	0,681	
	EDUCAT ⇒ ITEM3	0,08004	0,02788	0,02539	0,1347	0,09605	2.871	0,004	
	ITEM3 ⇒ CONVENIENCE	0,01856	0,02252	-0,0256	0,0627	0,04234	0,824	0,41	
	EDUCAT ⇒ ITEM4	0,0725	0,0303	0,01312	0,13188	0,08018	2.393	0,017	
	ITEM4 ⇒ CONVENIENCE	-0,0427	0,01885	-0,0796	-0,0057	-0,1056	-2.263	0,024	
Direct	EDUCAT ⇒ CONVENIENCE	0,01609	0,01245	-0,0083	0,04048	0,04404	1.292	0,196	
Total	EDUCAT ⇒ CONVENIENCE	0,01906	0,01227	-0,005	0,04311	0,05217	1.553	0,12	
**Type**	**Effect**	**Estimate**	**SE**	**Lower**	**Upper**	**β**	**z**	**P**	**%**
Indirect	EDUCAT ⇒ ITEM5 ⇒ CONVENIENCE	-0,0014	0,00116	-0,0037	8,50E-04	-0,0039	-12.256	0,22	**-0,07**
	EDUCAT ⇒ ITEM6 ⇒ CONVENIENCE	-0,0019	0,00141	-0,0046	8,88E-04	-0,0051	-13.292	0,184	**-0,10**
	EDUCAT ⇒ ITEM7 ⇒ CONVENIENCE	0,00427	0,0022	-4,77e−5	0,00859	0,01169	19.383	0,053	**0,22**
	EDUCAT ⇒ ITEM8 ⇒ CONVENIENCE	-2,79e−4	0,0024	-0,005	0,00443	-7,64e−4	-0,1162	0,908	**na**
	EDUCAT ⇒ ITEM9 ⇒ CONVENIENCE	0,00193	0,0018	-0,0016	0,00546	0,00528	10.722	0,284	**0,10**
	EDUCAT ⇒ ITEM10 ⇒ CONVENIENCE	0,00606	0,00256	0,00104	0,01108	0,01658	23.646	0,018	**0,32**
	EDUCAT ⇒ ITEM11 ⇒ CONVENIENCE	-3,37e−5	0,00215	-0,0042	0,00417	-9,22e−5	-0,0157	0,987	**na**
	EDUCAT ⇒ ITEM12 ⇒ CONVENIENCE	0,00887	0,00295	0,00309	0,01465	0,02429	30.084	0,003	**0,47**
Component	EDUCAT ⇒ ITEM5	0,0531	0,02822	-0,0022	0,10842	0,06312	18.815	0,06	
	ITEM5 ⇒ CONVENIENCE	-0,0267	0,01653	-0,0591	0,0057	-0,0615	-16.153	0,106	
	EDUCAT ⇒ ITEM6	0,05943	0,02473	0,01096	0,10789	0,08051	24.030	0,016	
	ITEM6 ⇒ CONVENIENCE	-0,0315	0,01974	-0,0702	0,00719	-0,0636	-15.956	0,111	
	EDUCAT ⇒ ITEM7	0,08824	0,02462	0,03999	0,1365	0,11963	35.845	<,001	
	ITEM7 ⇒ CONVENIENCE	0,04838	0,021	0,00723	0,08954	0,0977	23.043	0,021	
	EDUCAT ⇒ ITEM8	0,18483	0,03462	0,11698	0,25269	0,17665	53.390	<,001	
	ITEM8 ⇒ CONVENIENCE	-0,0015	0,01299	-0,027	0,02395	-0,0043	-0,1162	0,907	
	EDUCAT ⇒ ITEM9	0,13794	0,03494	0,06946	0,20641	0,13156	39.480	<,001	
	ITEM9 ⇒ CONVENIENCE	0,01399	0,01256	-0,0106	0,03861	0,04016	11.141	0,265	
	EDUCAT ⇒ ITEM10	0,09574	0,0191	0,05831	0,13318	0,16615	50.126	<,001	
	ITEM10 ⇒ CONVENIENCE	0,06327	0,0236	0,01703	0,10952	0,09981	26.817	0,007	
	EDUCAT ⇒ ITEM11	0,11658	0,02717	0,06333	0,16982	0,14277	42.912	<,001	
	ITEM11 ⇒ CONVENIENCE	-2,89e−4	0,01842	-0,0364	0,03581	-6,46e−4	-0,0157	0,987	
	EDUCAT ⇒ ITEM12	0,13187	0,03124	0,07064	0,19309	0,14049	42.212	<,001	
	ITEM12 ⇒ CONVENIENCE	0,06729	0,01569	0,03654	0,09804	0,1729	42.886	<,001	
Direct	EDUCAT ⇒ CONVENIENCE	0,00153	0,01226	-0,0225	0,02557	0,00419	0,1247	0,901	
Total	EDUCAT ⇒ CONVENIENCE	0,01906	0,01227	-0,005	0,04311	0,05217	15.533	0,12	

PCA: reduction of items on the 2020 data series (N= 885)[20], and reliability tests

a) entering19 items overall

Kaiser-Meye- Olkin Measure of Sampling Adequacy. 0,834 - Bartlett's Test of Sphericity ChiSquare 5677,360 P=0,00

Component	**Total Variance Explained** Initial Eigenvalues		
	Total	% of Variance	Cumulative %
1	4,351	22,899	22,899
2	2,901	15,269	38,168
3	2,318	12,200	50,369
4	1,153	6,069	56,438
5	1,021	5,376	61,814
6	0,859	4,520	66,334
7	0,773	4,070	70,404
8	0,690	3,632	74,036
9	0,618	3,254	77,291
10	0,606	3,188	80,478
11	0,556	2,924	83,402
12	0,528	2,776	86,179
13	0,501	2,635	88,814
14	0,451	2,372	91,186
15	0,427	2,250	93,435
16	0,365	1,922	95,357
17	0,322	1,696	97,053
18	0,283	1,488	98,541
19	0,277	1,459	100,000

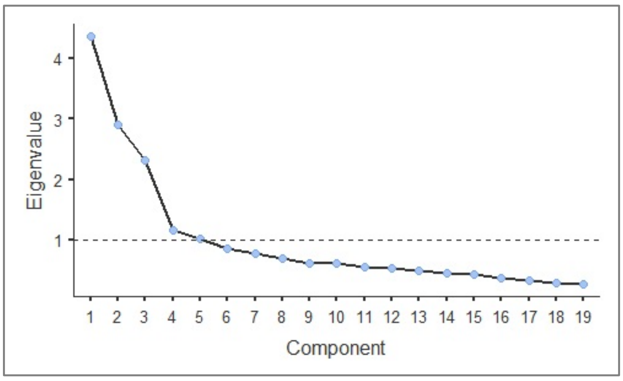

Fit measures			RMSEA 90% CI	
CFI	SRMR	RMSEA	Lower	Upper
0.919	0.0525	0.0589	0.0540	0.0639

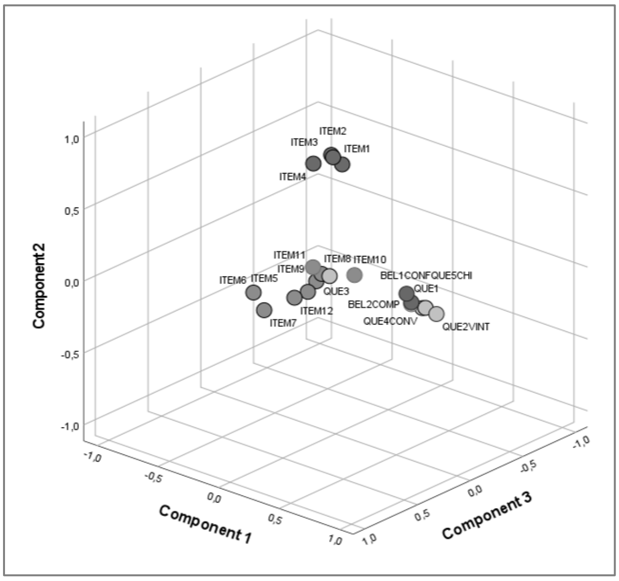

Component plot in rotated space

Cronbach's α 0.785 McDonald's ω 0.806

Item Reliability Statistics	If item dropped	
	Cronbach's α	McDonald's ω
ITEM1	0.756	0.791
ITEM2	0.748	0.787
ITEM3	0.752	0.789
ITEM4	0.756	0.791
ITEM5	0.756	0.790
ITEM7	0.747	0.783
ITEM8	0.761	0.793
ITEM9	0.770	0.796
ITEM10	0.757	0.788
ITEM11	0.743	0.782
ITEM12	0.741	0.779
QUE1	0.766	0.787
INTENTION	0.763	0.777
QUE3	0.766	0.796
CONVENIENCE	0.762	0.782
QUE5CHI	0.764	0.783
CONFIDENCE	0.755	0.775
COMPLACENCY	0.760	0.780

b) entering six items overall

Kaiser-Meyer-Olkin Measure of Sampling Adequacy.0,744 - Bartlett's Test of Sphericity Chi-Square 1393,236, P=0,00

Component	**Total Variance Explained** Initial Eigenvalues		
	Total	% of Variance	Cumulative %
1	2.719	45.31	45.3
2	1.087	18.11	63.4
3	0.837	13.95	77.4
4	0.651	10.85	88.2
5	0.385	6.42	94.6
6	0.321	5.35	100.0

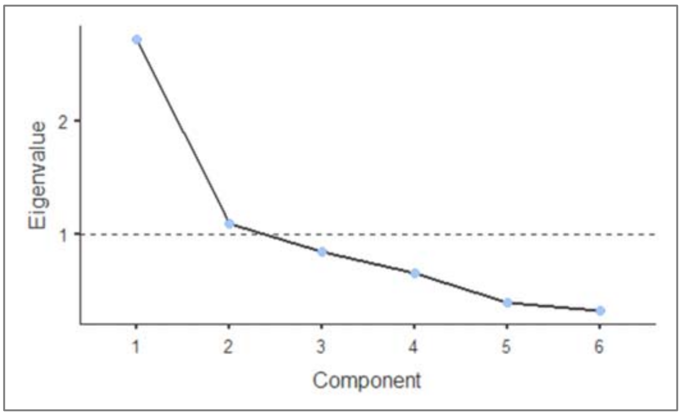

Cronbach's α 0.602 McDonald's ω 0.749

Item Reliability Statistics	If item dropped	
	Cronbach's α	McDonald's
ITEM2	0.647	0.787
ITEM12	0.623	0.763
INTENTION	0.550	0.671
CONVENIENCE	0.553	0.710
CONFIDENCE	0.474	0.663
COMPLACENCY	0.504	0.677

Fit Measures			RMSEA 90% CI	
CFI	SRMR	RMSEA	Lower	Upper
0.984	0.0221	0.0653	0.0425	0.0902

Fit measures[50]		acceptable fit
Comparative Fit Index	CFI	>.95
Standardized Root Mean Square Residual	SRMR	< .08
Root Mean Square Error of Approximation	RMSEA	< .08 or < .05

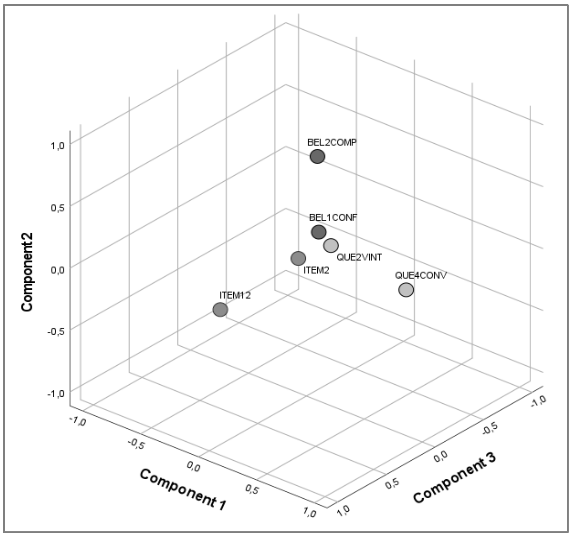

Component plot in rotated space
